# Transcriptome Sequencing of Tumor Subpopulations Reveals a Spectrum of Therapeutic Options for Squamous Cell Lung Cancer

**DOI:** 10.1371/journal.pone.0058714

**Published:** 2013-03-20

**Authors:** Christian L. Barrett, Richard B. Schwab, HyunChul Jung, Brian Crain, Daniel J. Goff, Catriona H. M. Jamieson, Patricia A. Thistlethwaite, Olivier Harismendy, Dennis A. Carson, Kelly A. Frazer

**Affiliations:** 1 Moores UCSD Cancer Center, University of California San Diego, La Jolla, California, United States of America; 2 Department of Pediatrics and Rady Children's Hospital, University of California San Diego, La Jolla, California, United States of America; 3 Bioinformatics and Systems Biology Graduate Program, University of California San Diego, La Jolla, California, United States of America; 4 Clinical and Translational Research Institute, University of California San Diego, La Jolla, California, United States of America; 5 Division of Cardiothoracic Surgery, University of California San Diego, La Jolla, California, United States of America; 6 Institute for Genomic Medicine, University of California San Diego, La Jolla, California, United States of America; 7 Department of Medicine, Stem Cell and Moores Cancer Center, University of California San Diego, La Jolla, California, United States of America; 8 Sanford Consortium for Regenerative Medicine, La Jolla, California, United States of America; Boston University Medical Center, United States of America

## Abstract

**Background:**

The only therapeutic options that exist for squamous cell lung carcinoma (SCC) are standard radiation and cytotoxic chemotherapy. Cancer stem cells (CSCs) are hypothesized to account for therapeutic resistance, suggesting that CSCs must be specifically targeted. Here, we analyze the transcriptome of CSC and non-CSC subpopulations by RNA-seq to identify new potential therapeutic strategies for SCC.

**Methods:**

We sorted a SCC into CD133− and CD133+ subpopulations and then examined both by copy number analysis (CNA) and whole genome and transcriptome sequencing. We analyzed The Cancer Genome Atlas (TCGA) transcriptome data of 221 SCCs to determine the generality of our observations.

**Results:**

Both subpopulations highly expressed numerous mRNA isoforms whose protein products are active drug targets for other cancers; 31 (25%) correspond to 18 genes under active investigation as mAb targets and an additional 4 (3%) are of therapeutic interest. Moreover, we found evidence that both subpopulations were proliferatively driven by very high levels of c-Myc and the TRAIL long isoform (TRAIL_L_) and that normal apoptotic responses to high expression of these genes was prevented through high levels of Mcl-1_L_ and Bcl-x_L_ and c-Flip_L_—isoforms for which drugs are now in clinical development. SCC RNA-seq data (n = 221) from TCGA supported our findings. Our analysis is inconsistent with the CSC concept that most cells in a cancer have lost their proliferative potential. Furthermore, our study suggests how to target both the CSC and non-CSC subpopulations with one treatment strategy.

**Conclusions:**

Our study is relevant to SCC in particular for it presents numerous potential options to standard therapy that target the entire tumor. In so doing, it demonstrates how transcriptome sequencing provides insights into the molecular underpinnings of cancer propagating cells that, importantly, can be leveraged to identify new potential therapeutic options for cancers beyond what is possible with DNA sequencing.

## Introduction

Lung cancer accounts for 28% of all cancer deaths—the highest percentage of all cancers [1]. Non-small cell lung cancer (NSCLC) accounts for ∼85–90% of lung cancers, of which adenocarcinoma and squamous cell carcinoma are the most common subtypes [1]. Although upwards of 70% of NSCLC patients have advanced disease that is rarely curable when diagnosed, new advances for subsets of lung adenocarcinomas that harbor EGFR mutations or EML4-ALK gene fusions encourage the development of targeted therapies that may alter this dire situation [2]. These genetic alterations primarily occur in adenocarcinomas of patients who never smoked, and are uncommon in SCC which is predominantly associated with smoking [3]–[5]. While FGFR1 [6] and DDR2 [7] have recently emerged as potential therapeutic targets for some SCC patients, inhibitors have yet to reach clinical trials. Recent NSCLC high throughput sequencing studies primarily focused on analyzing DNA have shown that few genes are mutated at a sufficiently high frequency to be useful for targeted therapy; however these studies do predict DNA alterations that are frequently clustered in a limited number of important molecular pathways suggesting that targeting these pathways may be a viable therapeutic strategy [8]–[12]. Deep transcriptome (RNA-seq) profiling of NSCLC to identify genes with deregulated expression that is common between tumors has not yet been reported, although such reports are to be expected given the large RNA-seq datasets being generated by TCGA [13] and other consortia.

Cancer cells within an individual tumor exist in distinct phenotypic states that often exhibit important functional differences. A subpopulation of cells with self-renewing and tumor-initiating capabilities, commonly referred to as cancer-stem-like cells (CSCs), have been identified in a variety of tumor types including NSCLC [14]. Mounting evidence suggests that CSCs are resistant to anticancer therapies and underlie metastasis [15], [16], and hence are the primary cancer cell type responsible for relapse and progression of malignant tumors. The immediate implication is that by targeting CSCs it should be possible to eradicate the drug resistant and metastatic subpopulation of a cancer [14]. However, recent studies have demonstrated that the CSC phenotype is plastic and can be reconstituted by other, non-CSC, tumor cells [17], [18]; thus not just CSCs but all tumor subpopulations that are “potential CSCs” must be targeted. Transcriptome sequencing of CSC and non-CSC subpopulations in NSCLC would provide insights into the molecular basis underlying their phenotypic similarities and differences and facilitate the identification of novel therapeutic targets. Such analysis will be an important and necessary complement to the bulk tumor transcriptome profiling being performed by TCGA and others.

The observations that non-CSCs can reconstitute CSCs, and vice versa, suggest that the phenotypic differences between these subpopulations are due to epigenetic rather than genetic differences. Therefore, exome and genome sequencing experiments aimed at identifying somatic mutations are not expected to reveal differences between sorted CSC and non-CSC subpopulations. On the other hand, transcriptome profiling, which is a readout of the epigenome (i.e. histone marks and DNA methylation that regulate expression), should be an excellent method for profiling CSCs and non-CSCs to reveal mechanistic differences. The advantage of RNA-seq data over microarrays is the ability to analyze isoform expression differences [19]. In cancer cells, alternative mRNA isoforms can produce protein isoforms with dominant negative activity. The pathogenic role of cancer-specific isoforms has been extensively demonstrated across all aspects of cellular physiology, including cellular adhesion and metastasis (CD44 and RON), cell growth and tumorigenesis (PKM2, MDM2, FGFR2, CRK, NUMB), cell cycle (PYK), angiogenesis (VEGF), apoptosis (GS3KB, CD95, Bcl-X, caspase-2, caspase-9), metabolism (PK), and drug resistance (AR and MRP-1) [20]–[24]. These examples underscore the advantage of isoform-level transcriptome information over whole gene expression for gaining insights into the molecular mechanisms underlying CSC and non-CSC phenotypic differences.

**Figure 1:**
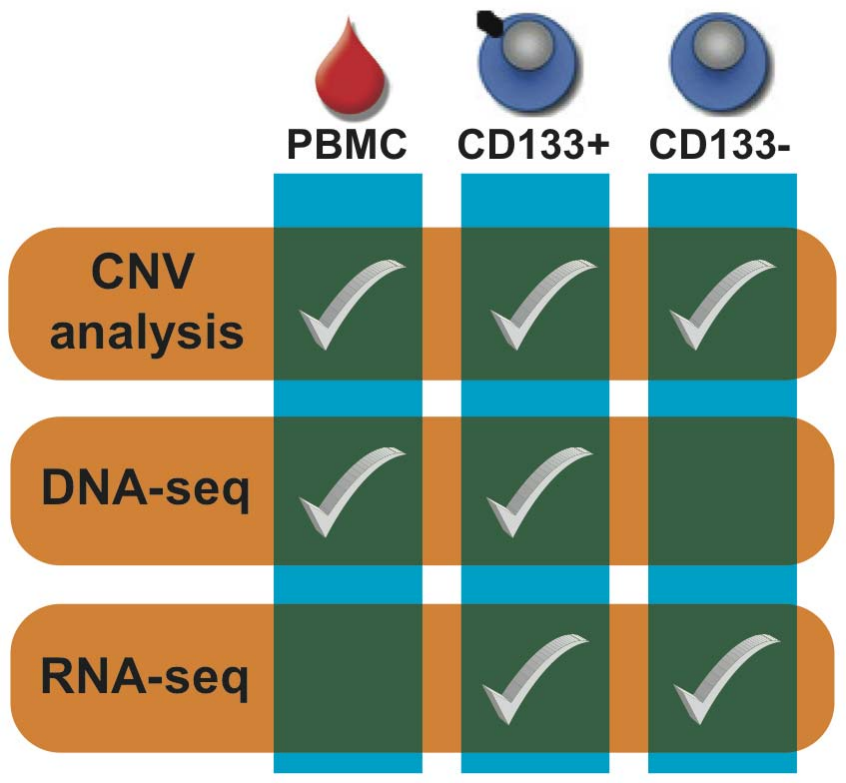
Study design. We sorted the human tumor cells of a squamous cell lung cancer xenograft into CD133+ and CD133− subpopulations. We then performed CNA analysis using genotyping arrays on peripheral blood mononuclear cells (PBMC) as well as both the CD133+ and CD133− subpopulations. We compared the genomes of the CD133+ population and PBMC sample to identify somatic mutations. Finally, we performed whole transcriptome sequencing (RNA-seq) of the CD133+ and CD133− subpopulations to evaluate their mRNA isoform expression differences and similarities.

Here we report the application of genomics technologies to a SCC xenograft that was sorted into CSC and non-CSC subpopulations based on the CD133 marker (Figure 1). CD133 (PROM1;Prominin-1) is a 5-transmembrane glycoprotein that is considered to be a marker for the subpopulation of CSCs in both subtypes of NSCLC [15], [25]–[27]. In NSCLC the CD133+ subpopulation has been shown to have higher tumorigenic potential in SCID mice, to express higher levels of stemness genes and to be more resistant to conventional chemotherapy than the CD133− subpopulation [15]. Importantly, so that the SCC xenograft would be more representative of primary tumor, it was directly engrafted as minced primary tumor into NSG mice and was never grown *in vitro*. Whole-genome DNA analysis revealed that the chromosomes of CD133+ and CD133− subpopulations were highly deranged in a very similar manner; however, as expected the tumor did not harbor clinically actionable mutations. Analysis of mRNA splice isoform expression profiles of the CD133+ and CD133− subpopulations resulted in the identification of SCC as a potential new indication for numerous drugs currently in development and suggest several additional new promising targets. Finally, analysis of The Cancer Genome Atlas (TCGA) publicly available transcriptome RNA-seq data of 221 SCCs [28] supports the generality of our transcriptome findings for this disease. Altogether our study demonstrates the capability of transcriptome sequencing of sorted cancer cell subpopulations to inform clinical development in ways that are not possible with DNA sequencing.

## Methods

### Xenografting

A UCSD Human Research Protections Program Institutional Review Board (IRB) approved this study prior to any study-related activities. All animal experimental procedures complied with the Guide for the Care and Use of Laboratory Animals (Institute for Laboratory Animal Research, 1996) and were approved by the Pfizer Global Research and Development Institutional Animal Care and Use Committee. A 75-year-old man with a 30-pack-year history of smoking and an incidentally found lung nodule provided written documented informed consent according to this IRB approved protocol prior to surgery. Resection revealed a T1 moderately differentiated squamous cell carcinoma. A portion of his resected fresh tumor was taken for xenografting into NSG (exact strain name NOD.Cg-Prkdcscid Il2rgtm1Wjl/SzJ) mice (The Jackson Laboratory). Subsequent passages were made in CB17.Cg-PrkdcscidLystbg/Crl mice from Charles River. Minced fresh tumor was mixed with Matrigel (BD Biosciences) and inserted subcutaneously. Mice were observed until palpable tumors grew and these were harvested for serial implantation and study. At two-year follow up the subject remained without clinical or radiographic evidence of relapse.

### Isolation of RNA and DNA

Lysates from the CD133−/EpCAM+ and CD133+/EpCAM+ sorted samples were processed for RNA and DNA extraction using Qiagen's All Prep Mini Kit (Valencia, CA). Samples were processed according to manufacturer recommendations. No more than four volumes were loaded on a single RNeasy Mini Spin column. Total RNA samples were evaluated for purity and concentration using the Agilent 2100 Bioanalyzer at the Genechip Microarray Core.

### RNA sequencing

Total RNA from both the CD133−/EpCAM+ and CD133+/EpCAM+ tumor subpopulations (∼230 ng each) were used to generate whole transcriptome libraries for sequencing on the SOLiD platform following manufacturer's recommendation (Life Technologies, Carlsbad CA, USA). The RNA samples were fragmented by RNAse III and concentrated using RiboMinus concentration Module (Invitrogen, Carlsbad CA, USA). RNA fragments were ligated to SOLiD adaptor mixes and reverse transcription performed. Purified cDNAs were size selected (150–250 bp) using the MinElute PCR purification kit (Qiagen Inc., Valencia, CA, USA), and then amplified 15 cycles using SOLiD 3′ and SOLID 5′ primers. Amplified cDNA was purified using PureLink PCR Micro kit (Invitrogen, Carlsbad CA, USA), QC'ed by the Bioanalyzer 2100 DNA 1000 kit (Agilent, Santa Clara, CA, USA), and quantified using the Qubit 2.0 Fluorometer (Invitrogen, Carlsbad CA, USA). The whole transcriptome libraries were used for making SOLiD templated beads following the SOLiD Templated Bead Preparation Guide and sequenced using the 50×25 paired-end protocol, for each sample generating more than 500 M read pairs per sample.

### Computational analysis of RNA-seq data

Transcriptome sequencing of the CD133+ and CD133− subpopulations produced 529 M and 531 M 50×25 read pairs, respectively. We first aligned the whole transcriptome paired-end reads to rRNA and tRNA genes using version 0.6.4 f of bfast+bwa [29] and retained only those pairs in which neither read was aligned, resulting in data sets comprising 77 M and 79 M read pairs. We then aligned each set of retained read pairs with bfast+bwa to a custom non-redundant database of mRNA isoforms constructed with the “cuffcompare” program from the version 0.9.3 of the Cufflinks software suite(79) and all isoform models in the RefSeq [30], UCSC Known Genes [31], and Ensembl [32] databases. We next converted the read alignment coordinates in the mRNA isoforms database to hg19 genomic coordinates using a custom script. From these alignments we computed expression and differential expression statistics with the “cufflinks” and “cuffdiff” programs of Cufflinks, respectively. When using both programs we used upper quartile normalization and the hg19 human genome reference sequence for bias correction. We computed that 13,612 genes and 43,120 gene isoforms had some non-zero level of expression in at least one of the two subpopulations. Figure S1 in Information S1 shows a histogram of these pre-filtered isoform expression values in both cell subpopulations. A majority of these instances were due to read mapping noise or to “leaky” gene expression that is of unknown physiological significance [33], so we applied minimum expression and read coverage criteria (>1 FPKM and 60% coverage) to obtain an initial isoform expression estimates in the two subpopulations. We observed that 3,844 genes with 7,558 mRNA isoforms met these minimal criteria. To be highly confident in our expression and differential expression results, we applied stringent filtering to the initial isoform expression estimates; we limited our analysis to mRNA isoforms that were expressed at a level of at least 30 FPKM in one of the subpopulations and which were covered over at least 60% of their length by sequencing reads. Additionally, for an isoform to be called differentially expressed, its differential expression had to be called significant at an FDR of 0.01 and to have changed at least four-fold between the two subpopulations. Application of these criteria yielded 572 genes had at least one isoform that was significantly differentially expressed. As a final result, we computed 671 of the 43,120 isoforms (1.6%) to be significantly differentially expressed. Table S1 in Information S1 shows how these numbers change for different FPKM and fold change criteria.

## Results

### Isolation of CSC and non-CSC subpopulations

**Figure 2:**
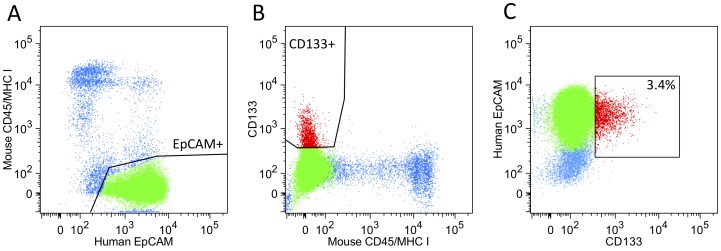
FACS isolation of CSCs (CD133+) and non-CSCs (CD133−) populations from a human squamous cell lung cancer xenograft. (A) Live cells displayed distinct populations of either mouse CD45/MHC I or human EpCAM expression. (B) Isolation of human CD133+ cells (red) from human CD133− cells (green) and mouse positive cells (blue). (C) Reanalysis of cell populations in panel B shows that the C133+ cells (red) are 3.4% of the EpCAM+ population.

We isolated human CSCs (CD133+/EpCAM+) and non-CSCs (CD133−/EpCAM+) from a squamous cell lung cancer xenograft using fluorescence-activated cell sorting (FACS) and respectively collected a total of 9.19×10^4^ (3.4%) and 2.18×10^6^ (96.6%) live cells corresponding to the two subpopulations (Figure 2). To assess the quality of our sort we measured expression levels of CD133 isoforms (Figure S2 in Information S1) in the whole transcriptome data (described below) and observed moderate expression of three isoforms in the CD133+ subpopulation and no detectable level of expression in the CD133− subpopulation. We performed a second FACS for the same xenograft isolated from a different animal and obtained a similar number and proportion of CD133+ and CD133− cells (Figure S3 in Information S1). Our results show that we are able to sort into pure CD133+ and CD133− subpopulations and that the CD133+ subpopulation constitutes a minor fraction of the cancer cells in the squamous cell lung tumor, which is consistent with previously published studies [15], [26].

### The mutational landscape of squamous cell lung tumor subpopulations

We performed Copy Number Alteration (CNA) analysis by microarray (see Results and Methods in Information S1) of germline, CD133+, and CD133− DNA and found that approximately half of the entire genome in both the CD133− and CD133+ subpopulations was involved in large CNAs (Figure S4 in Information S1). Overlap analysis revealed the potential existence of CNAs specific to each subpopulation, but upon manual inspection of all CNAs and taking into account the accuracy of microarrays [34], we could not confidently identify differences. Thus we concluded that the chromosomes of the CD133+ and CD133− subpopulations were highly altered in a largely indistinguishable manner. Additionally, we analyzed whole genome sequence data of the CD133+ subpopulation and matching germline DNA and concluded that the tumor studied did not contain any clinically actionable mutations (Tables S1, S2, S3, S4, S5, S6, S7, S8, S9, S10, S11, S12 in Information S2 and Figure S8 in Information S1).

### Evaluation of mRNA isoforms of genes encoding cell surface proteins

**Figure 3:**
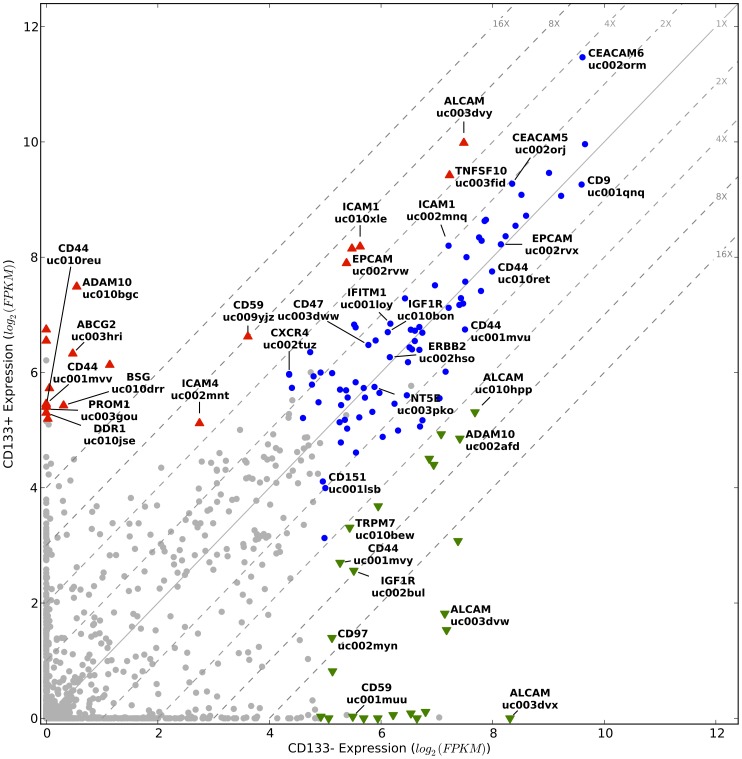
Isoform expression of cell surface genes in CD133+ and CD133− subpopulations. Displayed are the expression levels of 1,191 isoforms of 426 CD Molecule, 354 GPCR and 212 ion channel genes. Grey circles correspond to isoforms that did not meet our minimum expression level criteria and blue circles to isoforms that did but were not significantly differentially expressed. Colored triangles indicate significantly differentially expressed isoforms; green down-pointing triangles indicate decreased expression and red up-pointing triangles indicate increased expression in the CD133+ cells. The diagonal grey lines are fold change clines. Isoforms discussed in the text are named. Note that CD133/PROM1 does not meet our expression criterion, underlying the stringency of our data filtering; but is shown for comparison. Here we use HGNC-approved gene symbols [96] and UCSC isoform designations [31].

Deregulated expression of mRNA isoforms can result in the generation of cancer-associated cell surface proteins that are potential antigenic targets for therapeutic monoclonal antibodies. To identify such targets, we measured the expression of 1,191 mRNA isoforms of 426 CD Molecule [35], 354 GPCR [36], and 212 ion channel [36] genes (992 total genes). We limited our analysis to isoforms that were at least moderately expressed (30 FPKM) and covered over at least 60% of their length by sequencing reads in either or both the CD133+ and CD133− subpopulations, yielding an evaluation set of 124 isoforms representing 80 genes (10.4% of the 1,191 isoforms and 8.0% of the 992 genes) (Figure 3; Figures S5A, S5B in Information S1). Of the 124 highly expressed isoforms, 43 (in 25 genes) have at least four-fold expression differences (FDR<0.01), with 24 showing decreased and 19 showing increased expression in CD133+ cells relative to CD133− cells (Figure 3). Interestingly, 31 (25%) of the 124 highly expressed isoforms correspond to 18 genes currently under active investigation as drug targets for numerous cancers (specifically ABCG2 [37], ADAM10 [38], ALCAM [39], [40], BSG [41], CD151 [42], CD44 [43], CD47 [44], [45], CD9 [46]–[50], CEACAM5 [51], [52], CEACAM6 [52], CXCR4 [53], EPCAM [54], ERBB2 [55], ICAM1 [56], IGF1R [57], NRP1 [58], NT5E [59], TRPM7 [60]. Of note, the ABCG2 isoform (16X higher expression in the CD133+ subpopulation) and the CXCR4 isoform (4X higher expression in the CD133+ subpopulation) have previously been identified as biomarkers of lung cancer tumor initiating cells spared by cisplatin treatment [15]. In addition to these 18 genes under active investigation as drug targets two other highly expressed genes—CD97 [61], and IFITM1 [62]—have been proposed but not actively pursued as therapeutic antibody targets for primary or metastatic tumors. We believe that other highly expressed cell-surface genes shown in Figure 3 may also be potential drug targets. For example, an isoform of DDR1, a homolog of DDR2 that is a promising target for SCC [7], is highly expressed in CD133+ cells. ICAM4, which has a demonstrated role in carcinogenesis and is located in the 19p13.2 susceptibility locus for multiple cancers [63], [64], is also highly expressed. As these 22 cell surface genes of therapeutic interest encode for proteins involved in cell-cell interactions, cell adhesion, migration, and chemoresistance, their expression similarities and differences identified here illustrates how isoform-level analyses can provide a new level of information for both understanding the physiology of, and for targeting, specific tumor subpopulations.

**Figure 4:**
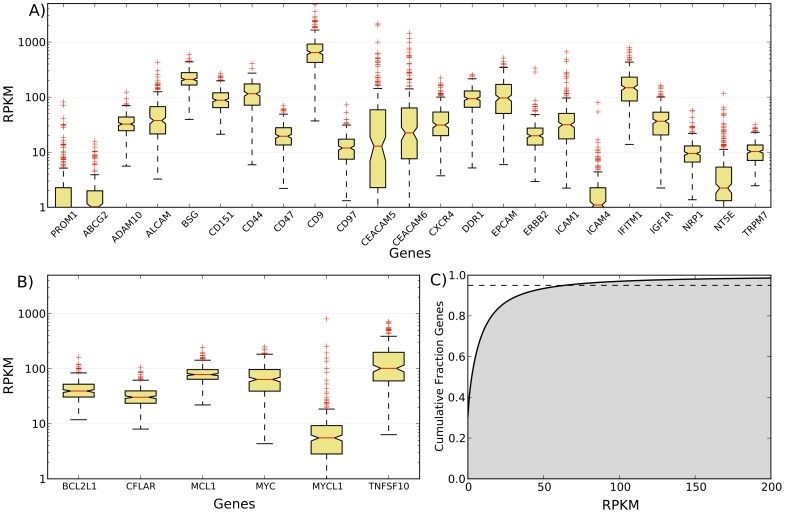
Box plots of whole gene expression measurements from TCGA lung squamous cell carcinomas. A) Cell surface and B) apoptosis-related whole gene expression estimates from 221 lung squamous cell carcinoma samples. C) The distribution of expression levels estimated for 20,533 genes per sample, averaged across all 221 samples. The dotted horizontal line indicates the top 5% of genes, when ranked by expression.

To assess the degree to which high expression of the therapeutically attractive cell surface proteins described above for the studied tumor is a common phenomenon in SCC we examined TCGA transcriptome data of 221 squamous cell lung cancers [28], [65]. Importantly, TCGA sequencing is performed on bulk tumor, so the subpopulation composition is expected to be quite different than the CD133+ and CD133− subpopulations of the squamous cell carcinoma in our study. The publicly available TCGA expression data is analyzed at the exon-, splice junction-, and gene-level. Since there is no established way to infer isoform-level expression from exon and splice junction expression, we chose to compare our isoform level expression estimates with TCGA gene level expression estimates. Based on the fact that our analysis focused on isoforms with high expression, we reasoned that if our findings are general to SCC then we would observe high expression of the corresponding whole genes across a majority of the 221 tumor samples. As shown in Figure 4A, 4C, the majority of the 22 cell surface genes of therapeutic interest are robustly expressed across all 221 samples with 12 placing in the top 10% (median expression >31 RPKM) of the most highly expressed genes. Interestingly, ABCG2 and ICAM4, which have the two lowest expression levels of the 22 genes, were predominantly expressed in the CD133+ subpopulation. This situation mirrors that of CD133/PROM1 (Figure 3), so we speculate that the apparently low tumor-wide expression of ABCG2 and ICAM4 is because they are robustly expressed only in CD133+ cells, which constitute a minor fraction of the tumor cells. Overall, these results indicate that our findings are general for SCC and so could impact clinical development because they identify this disease as a potential new indication for numerous drugs currently under development and suggest several additional new promising targets.

### Evaluation of mRNA isoforms of genes involved in apoptosis

**Figure 5:**
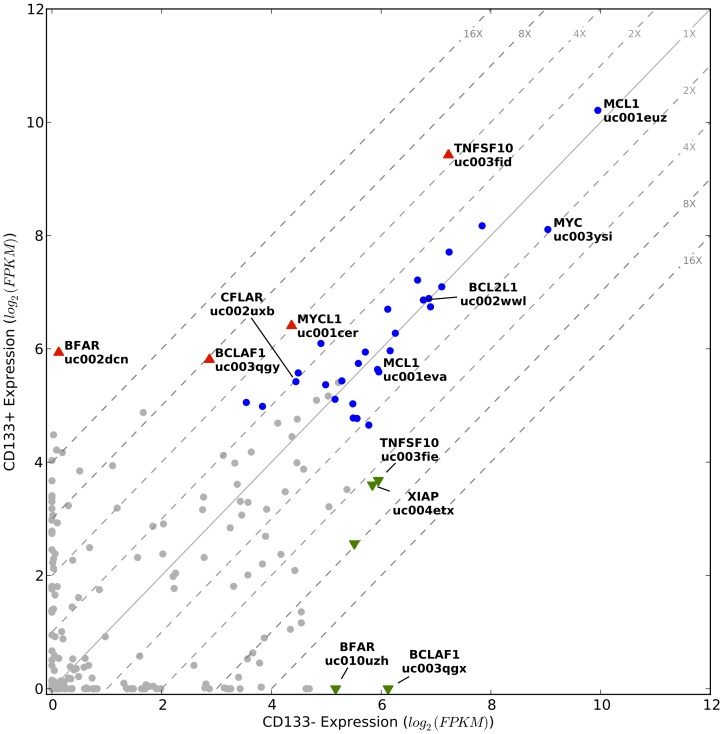
Isoform expression of apoptosis-related genes in CD133+ and CD133− subpopulations. Displayed are the expression levels of 434 isoforms of 106 apoptosis-related genes. Grey circles correspond to isoforms that did not meet our minimum expression level criteria and blue circles to isoforms that did but were not significantly differentially expressed. Colored triangles indicate significantly differentially expressed isoforms; green down-pointing triangles indicate decreased expression and red up-pointing triangles indicate increased expression in the CD133+ cells. The diagonal grey lines are fold change clines. Isoforms discussed in the text are named. Isoforms discussed in the text are named. Here we use HGNC-approved gene symbols [96] and UCSC isoform designations [31].

Given the extent of genomic alterations observed in the CNA analysis of the studied SCC and the multitude of known cell death mechanisms that respond to DNA damage, we suspected that the cancer cells must have altered expression of apoptosis-related genes in order to survive under such a high mutational load. To gain insights into the survival capacity of the CD133+ and CD133− subpopulations, we performed a focused expression analysis on a set of 106 apoptosis-related genes (Table S2 in Information S2) that altogether had 434 mRNA isoforms. As above, we limited our analysis to isoforms that were expressed at the level of at least 30 FPKM and that were covered over at least 60% of their length by sequencing reads in either or both of the subpopulations. Thirty of the 106 genes (28%) and 37 of the 434 isoforms (9%) met these criteria (Figure 5; Figure S6 in Information S1). Interestingly, only 9 isoforms in 6 genes were significantly differentially expressed with at least a four-fold difference between the two subpopulations (FDR<0.01). Noteworthy is the high expression of an L-Myc isoform (MYCL1,uc001cer) and an XIAP isoform (XIAP, ucoo4etx) in both subpopulations, with four-fold upregulation in the CD133+ and the CD133− subpopulations, respectively. L-Myc is a globally acting transcription factor and of all of the proteins in the Myc family (N-Myc, c-Myc, and L-Myc) it has the strongest and most specific activity in promoting human iPSC generation—presumably through its ability to suppress differentiation-associated genes [66]. XIAP is a well-known suppressor of apoptosis in addition to other physiological roles related to its E3 ubiquitin ligase activity [67]. Also noteworthy are the few isoforms (i.e. BCLAF1 and BFAR isoforms) with >16x differential expression. BCLAF1, which has an isoform highly expressed only in the CD133− subpopulation, plays critical roles in many processes [68], including lung development [69]. BFAR, an E3 ubiquitin ligase that likely mediates crosstalk between the intrinsic and extrinsic apoptotic pathways [70], has one isoform expressed exclusively in CD133+ and another exclusively in CD133−. These results suggest that, although overall the CD133+ and CD133− subpopulations similarly express apoptosis-related genes, expression differences of isoforms of key apoptosis genes may in part contribute to self-renewal and survival differences between these cancer cell types.

We next focused on the most highly expressed apoptosis-related genes in both the CD133+ and CD133− subpopulations to gain insights into survival mechanisms common between these cancer cell types. This focused analysis revealed c-Myc (MYC,uc003ysi), and the long isoforms of TRAIL/TNFSF10 (TNFSF10,uc003fid), Mcl-1 (MCL1,uc010pch), and Bcl-x (BCL2L1,uc002wwl) to be among the most highly expressed apoptosis-related genes (Figure 5). We confirmed by RT-qPCR the high expression of c-Myc, Mcl-1_L_, and Bcl-x_L_ in both the CD133+ and CD133− subpopulations (Figure S7 in Information S1). It is well established that high c-Myc expression potently induces the intrinsic (mitochondria-mediated) cell death pathway; however concurrent high expression of Mcl-1_L_ and Bcl-x_L_ can sequester the mitochondrial outer membrane proteins Bak and Bax [71], [72], and thereby block the apoptotic consequences of c-Myc. Such a role for Mcl-1_L_ and Bcl-x_L_ has been recently demonstrated in 14 c-Myc-driven non-squamous cell lung cancer human cell lines [73], in mouse models of adenocarcinoma lung cancer [74], and in other malignancy contexts [75]. Our data implicate Mcl-1_L_ and Bcl-x_L_ in blocking the apoptotic effects of c-Myc in both the CSC and the non-CSC subpopulation of the studied squamous cell carcinoma. We note that while the long isoforms of Mcl-1 and Bcl-x have demonstrated pro-survival functions, the short isoforms of these genes, which were not expressed, are pro-apoptotic [76]. High expression of the long isoform of TRAIL (TRAIL_L_) in both subpopulations is significant, as this cytokine is under investigation as an anti-cancer agent [77] because it can selectively kill tumor cells via receptor-mediated apoptosis. However, prolonged exposure to high levels of TRAIL_L_ can result in TRAIL_L_ resistance, after which TRAIL_L_ becomes a strong inducer of proliferation and metastasis [78]. Previous studies have shown both Mcl-1_L_, and Bcl-x_L_ to be responsible for acquired TRAIL_L_ resistance in non-squamous lung cancer cell lines [79] and in other cancer cell lines [80]–[83]. Of note, another major determinant of TRAIL resistance, c-Flip_L_ (CFLAR,uc002uxb)–the long isoform of Flip/CFLAR that is a catalytically inactive homolog of caspase-8–is also highly expressed in both subpopulations of our studied SCC (Figure 5) [79], [84]–[87]. The short isoforms of both TRAIL and c-Flip also have antithetical pro-apoptotic roles compared to the long isoforms [79], [88]. Altogether our isoform-level gene expression analysis suggests that in the studied SCC both the CD133+ and CD133− subpopulations were driven by very high levels of c-Myc and TRAIL_L_ and that the normal apoptotic response to the high expression of these genes was prevented through very high levels of Mcl-1_L_ and Bcl-x_L_ and c-Flip_L_.

Again we used TCGA transcriptome data of 221 squamous cell lung cancers to determine the generality of our apoptosis gene isoform analysis results. As above for ABCG2 and ICAM4, we attribute the apparently low tumor-wide expression of L-Myc (MYCL1) (Figure 4B, 4C) to the possibility that in SCC it is robustly expressed only in CD133+ cells, which constitute a minor fraction of the tumor cells. Myc (MYC), TRAIL (TNFSF10) and Mcl-1 (MCL1) are among the top 5% of most highly expressed genes (median expression >63 RPKM) and Bcl-x (BCL2L1) and c-Flip (CFLAR) are among the top 10% of most highly expressed genes across all 221 samples (Figure 4B, 4C). TCGA data does not provide mRNA isoform level expression estimates, though, which is crucial for accurate biological interpretation given the antithetical roles of the long and short isoforms of Mcl-1, Bcl-x, c-Flip, and TRAIL [89], [90]. Thus, our isoform analysis provides insights into the shared biological mechanisms underlying survival of both CSC and non-CSC subpopulations in the studied SCC and TCGA data support the generality of our findings.

## Discussion

Our study is an important contribution to the understanding of the potential of CSC-targeted therapy [14]. To our knowledge, it is the first to leverage transcriptome sequencing for the purpose of discovering potentially translatable therapeutic strategies based on CSCs. Indeed, few (if any) genomic investigations that leverage current understanding of the CSCs have been reported. There are two main reasons. First, the CSC concept posits that the difference between CSCs and non-CSCs is epigenetic and not genetic, so comparative DNA sequencing is not likely to yield any results that could not be obtained from bulk tumor sequencing. Comparative RNA sequencing, on the other hand, should yield new insights given the expected physiological differences between CSCs and non-CSCs. RNA-seq, though, is a relatively new technique and the needed informatics methods for transforming raw sequencing output into information that reliably reflects the complexity of the transcriptome (particularly isoform calling) are still under development.

While the use of CD133 as a CSC marker is an unresolved issue, our transcriptome measurements do indeed reveal pronounced gene usage in the CD133+ subpopulation that one would expect in a CSC population (e.g. L-Myc, ABCG2, CXCR4). But the expression differences of these genes' isoforms are a matter of degree and do not support a clear and definitive distinction between the two subpopulations—consistent with recent demonstrations of the plasticity of the CSC phenotype. Furthermore, our transcriptome measurements are inconsistent with the CSC concept tenet that a majority of the cells in a cancer have lost their proliferative potential; because c-Myc and TRAIL_L_ are very highly expressed in both CD133+ and CD133− cells, both subpopulations have high tumorigenic potential. Tumorigenic potential is the key consideration for defining a targeted therapy, so our analysis results suggest that essentially all of the cells in the studied tumor would need to be targeted, regardless of whether or not SCC harbors CSCs and whether or not CD133 is the most accurate way to identify them.

It is important to highlight that the extra expense and effort associated with transcriptome sequencing and cell sorting were necessary for obtaining our results. For instance, expression profiling of bulk tumor would have revealed high expression of c-Myc, L-Myc, Mcl-1, Bcl-x, c-Flip, and TRAIL, but (critically) would not have revealed which isoforms of Mcl-1, Bcl-x, c-Flip, and TRAIL were being expressed. Furthermore, if we had not sorted the tumor cells using what is currently the best marker of CSCs in SCC we would not have been able to identify phenotypic overlap between CSCs and non-CSCs and targetable mechanisms and proteins that could potentially eradicate all tumor cells.

For complete eradication of a malignancy, it is often presumed that separate therapies targeting the CSC and the non-CSC subpopulations will be required. Our results, on the other hand, suggest the possibility of targeting both subpopulations with one treatment strategy. Our analysis indicates that blocking the anti-apoptotic effects of Bcl-x_L_ and Mcl-1_L_ may render both the CSC and non-CSC subpopulations susceptible to c-Myc- and TRAIL_L_-induced apoptosis. Such a strategy could potentially be achieved using the BCL2 family pan-inhibitor Sabutoclax [91], currently being investigated for leukemias because of its ability to inhibit both Bcl-x_L_ and Mcl-1_L_ [92]. Our observation of high c-Flip_L_ expression suggests an even more robust strategy in which both subpopulations could be additionally sensitized to TRAIL_L_-induced apoptosis via the extrinsic cell death pathway by treatment with, for instance, triterpenoids [93], [94], troglitazone [95], or flavopiridol(69). While to our knowledge no therapies targeting both the CSCs and non-CSCs in a single type of malignancy have been articulated, others [92] have suggested that such a dual-target therapy might prove the most effective for tumor eradication because all tumor cells would be targeted—which is the safest approach given the distinct possibility that the CSC phenotype is a not a stable trait.

## Conclusions

We have used a SCC xenograft to demonstrate the power of transcriptome sequencing of CSC-marker-sorted subpopulations to: 1) identify SCC as a new potential indication for numerous ongoing drug development efforts; 2) illustrate how isoform level expression measurements can be used to inform the targeting of tumor subpopulations; and 3) gain insight into tumor physiology and develop a new therapeutic strategy with high clinical potential. Our study is relevant to SCC in particular for it presents numerous possible options to standard radiation and cytotoxic chemotherapy where none currently exist, and it is relevant to cancers in general because it demonstrates how leveraging the CSC concept and isoform-level transcriptome profiling can provide new insights into cancer therapy beyond what would be possible with DNA sequencing.

## Supporting Information

Information S1 **Supplemental Materials.**
(DOCX)

Information S2 **Supplemental Table.**
(XLSX)
