# Supplementary material for: Transcriptome Sequencing of Tumor Subpopulations Reveals a Spectrum of Therapeutic Options for Squamous Cell Lung Cancer
Source: PLoS One. 2013 Mar 20;8(3):e58714. doi: 10.1371/journal.pone.0058714 (PMC3604164; doi:10.1371/journal.pone.0058714)
Supplement: Information S1 — Supplemental Materials. (DOCX) [file pone.0058714.s001.docx]

*Supporting Online Material for*

**Transcriptome sequencing of tumor subpopulations reveals a spectrum of therapeutic options for squamous cell lung cancer**

**Authors**: Christian L. Barrett^1,2^, Richard B. Schwab^1,4^, HyunChul Jung^1,3^, Brian Crain^1^, Daniel J. Goff**^7^**, Catriona H. M. Jamieson**^7^**, Patricia A. Thistlethwaite^5^, Olivier Harismendy^1,2,4,^, Dennis A. Carson**^8^**, Kelly A. Frazer^1,2,4,6†^.

1. Moores UCSD Cancer Center, 2. Department of Pediatrics and Rady Children's Hospital and 3. Bioinformatics and Systems Biology graduate program and 4. Clinical and Translational Research Institute and 5. Division of Cardiothoracic Surgery and 6. Institute for Genomic Medicine, University of California San Diego, 9500 Gilman Drive, La Jolla CA 92093, 7. Department of Medicine, Stem Cell and Moores Cancer Center, University of California San Diego, 3855 Health Sciences Drive, La Jolla, 92098 CA USA, 8. Sanford Consortium for Regenerative Medicine, 2880 Torrey Pines Road, La Jolla, CA 92093

^†:^ Corresponding Author: [kafrazer@ucsd.edu](mailto:kafrazer@ucsd.edu)

**SUPPLEMENTAL RESULTS**

**The mutational landscape of squamous cell lung tumor subpopulations.**

We performed Copy Number Alteration (CNA) analysis by microarray of germline, CD133+, and CD133- DNA and found that approximately half of the entire genome in both cancer cell subpopulations was involved in large CNAs (Figure S4 in File S1). Overlap analysis revealed the potential existence of CNAs specific to each subpopulation, but upon manual inspection of all CNAs and taking into account the accuracy of microarrays (1), we could not confidently identify differences. Thus we concluded that the chromosomes of the CD133+ and CD133- subpopulations were highly altered in a largely indistinguishable manner.

A previous study identified recurrent large-scale genomic alterations on 26 autosomal chromosomal arms of 371 adenocarcinoma lung cancer samples (2). Many of these large-scale rearrangements are also present in the studied squamous cell lung tumor —including amplifications of 1q, 5p, 7p, 8q, and deletions of 3p, 5q, 6q, 8p, 9p, 9q, 13q, 15q, and 17p (Figure S8 in File S1). These results suggest that many of the large copy number alterations observed in the studied squamous cell carcinoma may be generalizable to other lung cancer histologies. To gain insight into the biological consequences of the CNAs, we examined 17 genes identified in the COSMIC database (3) as frequently mutated in lung cancer (Table S3 in File S2) and determined that 9 are in genomic regions deleted in the squamous cell carcinoma, 3 are in amplified intervals and 5 are in copy number neutral regions (Figure S8 in File S1). Thus the highly rearranged chromosomes observed affect the copy number status of many lung cancer genes and are consistent with the gross alterations characteristic of NSCLC.

We next analyzed whole genome sequence data of the CD133+ subpopulation and matching germline DNA to identify clinically actionable mutations (see Table S4 in File S2). Interestingly, none of the 17 NSCLC disease-relevant genes, including EGFR and KRAS, were identified as carrying somatic mutations. As anticipated, these results indicate that this squamous cell lung cancer subject would not have benefited from treatment with EGFR targeted inhibitors or monoclonal antibodies. To determine if anaplastic lymphoma kinase inhibition would have been a therapeutic option, we used the whole transcriptome sequencing data to look for evidence of an EML4-ALK translocation. We measured high EML4 expression but no ALK expression in both the CD133+ and CD133- subpopulations, thereby ruling out constitutive ALK activation in the studied tumor sample. Therefore DNA mutational analysis did not reveal potentially clinically actionable mutations.

**SUPPORTING METHODS**

*Cell Sorting.* The human squamous cell carcinoma NSCLC xenograft tumors were harvested and prepared for fluorescence-activated cell sorting (FACS) by mechanical and enzymatic disaggregated into single cell suspensions. Tumors were minced with a razor blade into small fragments (1-2mm^3^) to facilitate disaggregation. Minced tissue was incubated with Accumax (Innovative Cell Technologies) at 37**°**C in an orbital shaking water bath for 1 hour. Suspensions were successively strained through 100 and 40 μm cell strainers to remove debris. All antibody staining steps were performed in FACS Stain Buffer (BD Biosciences). To minimize nonspecific binding of antibodies, cells were first incubated with mouse CD16/CD32 (BD Biosciences) and human FC-block (Miltenyi). Suspensions were subsequently stained with the human epithelial marker EPCAM-APC (clone EBA-1; BD Biosciences) and CD133-PE (clone AC133; Miltenyi) to identify a human cells and obtain both CD133+ and CD133- subpopulations. Mouse cells were excluded by simultaneous staining with biotinylated anti-mouse CD45 (clone 30-F11; BD Biosciences) and anti-mouse H-2K^d^ (clone SF1-1.1; BD Biosciences) followed by streptavidin PE.Cy7 (BD Biosciences). Using a BD FACSAria cell sorter (Becton Dickinson, San Jose, CA) forward and side scatter area versus height signal plots were used to eliminate cell doublets. Dead cells were avoided by excluding propidium iodide positive cells, while contaminating mouse cells were eliminated by excluding PE-Cy7 positive cells. A total of 2.18×10^6^ and 9.19×10^4^ live human cells were collected corresponding respectively to the CD133-/EpCAM+ and CD133+/EpCAM+ subpopulations. Sorting was performed directly into RLT Plus Buffer containing 2-mercaptoethanol (Qiagen, Valencia, CA). Immediately following collection the volume was adjusted to 3.5 volumes of RLT to 1 volume of sorted sample.

*Genotyping Array Data Generation and* *Copy Number Variant analysis.* Germline DNA and both the CD133-/EpCAM+ and CD133+/EpCAM+ cancer cell subpopulations were assayed on Illumina HumanOmni1-Quad BeadChip kits according to manufacturer’s recommendations (Illumina, San Diego, CA, USA). CNV analysis was performed with the CnvPartition algorithm(73), which was run as a plug-in within the GenomeStudio browser with default settings (confidence threshold 35, minimum number of probes 3). CnvPartition algorithm models log R ratio (LRR) and B allele frequency (BAF) for each of fourteen different copy number scenarios as simple bivariate Gaussian distributions. Specifically, the likelihood of observing a given LRR and BAF under each of the fourteen models is calculated.

# There were ~500 called CNVs in both the CD133+ and CD133- subpopulations spanning ~1.4 Gb of the human genome compared to ~190 called CNVs spanning 4.4 Mb in the germline sample (Table S5 in File S2). We removed CNVs showing loss of heterozygosity (LOH) and merged adjacent CNVs (within 20kb) having the same copy number (ranging from 0 to 4 copies) into single CNVs; this resulted in modest changes in the number of called CNVs and total length of the human genome involved in CNVs. Approximately 95% of the CNVs (and 95% of the corresponding DNA bases) identified in CD133+ subpopulation overlap CNVs identified in the CD133- subpopulation and vice versa (Table S6 in File S2). The CNV concordance between the CD133+ and CD133- samples is higher than that observed for replicate samples in published comprehensive assessments of array-based platforms and calling algorithms for detection of CNVs(24). The genotyping statistics from the Illumina HumanOmni1-Quad BeadChip further support that the large segments of the human genome involved in CNVs are highly similar in the CD133+ and CD133- samples (Table S7 in File S2). These data indicate that the CNVs in the CD133+ and CD133- subpopulations are likely to be identical.

To identify genes of potential importance for NSCLC initiation and progression we downloaded the COSMIC list, updated 2010-03-30 (3) and created a list of 17 genes (with 52 associated isoforms) marked as having mutations in NSCLC or lung cancer (Table S3 in File S2).

*DNA sequencing.* Germline DNA (1.0 ug) and the CD133+/EpCAM+ tumor subpopulation DNA (200 ng) was directly used as starting material for SOLiD fragment library preparation (Life Technologies, Carlsbad CA, USA) following manufacturer’s recommendation. DNA was sheared to approximately 150 bp using the Covaris S2 system standard fragmentation conditions recommended in the SOLiD4 Library Prep User Guide. After DNA end-repair, P1 and P2 adaptors were ligated, the adaptor-ligated DNA underwent nick translation and then amplification with 6 and 8 PCR cycles for germline and tumor DNA respectively, using Library PCR primer 1 and 2, and Platinum PCR amplification mix. Purified library was quantified by TaqMan assay and used for preparing SOLiD templated beads. Each sequencing experiment generated ~500M 50-bp reads per slide. The samples were sequenced over several runs each using the SOLiD platforms generating more than 1.7 billion total raw reads per sample (Table S8 in File S2).

*Computational analysis of DNA sequencing reads.* We used Bioscope version 1.3.1 with default settings to perform sequence alignment of SOLiD color-space raw reads to the NCBI human reference genome (hg19). Initial alignments were refined by base quality recalibration and local re-alignment using GATK v2 (Genome Analysis Tool Kit)(4). To remove PCR duplicates we used Picard v1.42(5) because it retains the read with the highest mapping quality. After removal of duplicate reads the germline and CD133+ tumor subpopulation had a coverage depth respectively of 13X and 5X (Table S8 in File S2). Somatic variants were identified from the alignment data using Varscan v2.2.3 somatic module(6). To identify somatic single nucleotide variants (SNVs) we required that 1) they be supported by at least two reads in the tumor and 2) the variant frequency for a heterozygote to be greater than 20%. To call somatic indels, we required 1) the positions had to have a minimum coverage greater than 10 in both the tumor and germline samples and 2) clusters of false positives and SNV calls near indels to be removed before using the Varscan somatic filter.

To further reduce false positive calls, we removed variants with a low average alternate allele base quality score using the model based clustering algorithm, MCLUST(77). MCLUST selects the optimal model according to BIC (Bayesian Information Criterion) for EM (Expectation Maximization) initialized by hierarchical clustering for parameterized Gaussian mixture models. Based on the observed bimodal distribution of the average alternate allele base quality score of the novel variants, we ran MCLUST with the default parameters except for G = 2, where G is the number of clusters for which the BIC is to be calculated. Results consisted of two different z scores (Z_1_ and Z_2_) for each variant site; Z_1_ and Z_2_ being the probabilities of a novel variant belonging to the first cluster and the second cluster (Z_1_ = 1 – Z_2_). To find the minimum alternate allele base quality (AABQ) score for all novel variant sites we selected the minimum AABQ that had a z score of being in the second distribution (Z_2_) >= 0.95. The second distribution of the novel variant sites’ AABQ had a similar distribution to the AABQ of known variant sites; this is why we chose a probability of 0.95 of being in Z_2_ as the minimum z score. After calculating the minimum AABQ for novel variant sites we applied this minimum AABQ score to all variant sites, novel and known sites. Summary information on the germline variants, somatic single nucleotide and indel mutations is given in Tables S4,S8,S9 in File S2.

To perform our focused mutation analysis of disease-relevant genes, we first downloaded the known somatic mutation positions for the 17 known NSCLC genes from v54 of the COSMIC database(3). We first quantified the percentage of each gene in which we could call variants, given our criteria described above. As tabulated in Table S11 in File S2, these percentages ranged from 20-48% for entire genes (i.e. intronic plus exonic regions) and 27-92% for only exonic regions. The known “actionable” mutations in EGFR all reside in exons 18-21, which had a coverage rate ranging from 7-66% (Table S12 in File S2). While these ranges of coverage did not allow us to comprehensively survey the entire exons for new somatic mutations, we were able to accurately call point mutations at L858 and L861—which respectively account for ~45% and 3% of all EGFR mutations(7). Position G719, which accounts for 2% of all EGFR mutations, was insufficiently covered for variant analysis. KRAS, on the other hand, was sufficiently covered such that we could evaluate the mutational status of all nucleotides corresponding to G12, G13, and Q61 in the KRAS protein, which is recommended by the FDA for testing to predict how patients will respond to certain EGFR inhibitor drugs.

*Analysis of TCGA data.* We downloaded the Level 3 RNAseq analysis data for the 221 squamous cell lung (LUSC) tumors available from the TCGA (8) on April 16, 2012. To assess the distribution of the expression level of a gene of interest, we extracted its RPKM from the 221 whole gene expression text files. To determine the averaged cumulative fractional distribution of RPKM values across all 221 samples, we histogrammed the RPKM values of each of the 221 whole gene expression text files, normalized each histogram by the number of genes (20,533), computed the average histogram, and then computed the averaged histogram’s cumulative fractional distribution.

*Quantitative RT-PCR.* RNA isolated from the CD133-/EpCAM+ and CD133+/EpCAM+ tumor subpopulations (50ng each) was used to generated cDNA using SuperScript III First-Strand Synthesis SuperMix (Invitrogen, Carlsbad CA, USA) and quantitative PCR (qRT-PCR) was performed in duplicate on an iCycler (Bio-Rad) using SYBR GreenER Super Mix (Invitrogen, Carlsbad CA, USA). 5ng of template cDNA and 0.4mM of each forward and reverse primer was used per qRT-PCR reaction. The following primers were used: *MCL1_L_* Fwd/Rev: agaccttacgacgggttgg */* aatcctgccccagtttgtta, *BCLX_L_* Fwd/Rev: catggcagcagtaaagcaag / gaaggagaaaaaggccacaa*, cMYC* Fwd/Rev: acgtctccacacatcagcac / cgcctcttgacattctcctc, *RPL27* Fwd/Rev: atcgccaagagatcaaagataa */* tctgaagacatccttattgacg (not shown in Figure S7 of File S1). The specificity of the PCR reactions was confirmed by melting curve analysis and all products were verified by sequencing. mRNA levels for each transcript were normalized to RPL27 and compared between samples using the delta-delta CT method.

**SUPPORTING FIGURES**

**Figure S1:** Histograms of the expression values of all non-tRNA/rRNA mRNA isoforms before minimum expression and coverage filtering**.**


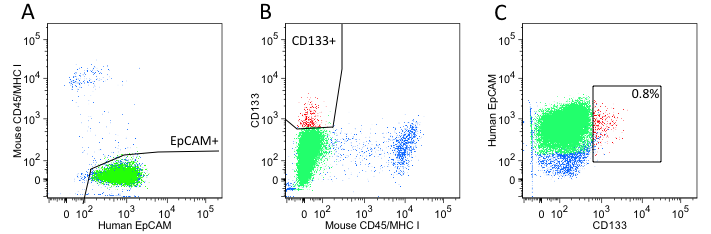


**Figure S3: FACS of the human SSC NSCLC xenograft obtained from a second animal**. (A) Live cells designated as propidium iodide negative displayed distinct populations of either mouse CD45/MHC I or human EpCAM expression. (B) Isolation of CD133+ cells from human CD133- cells and mouse positive cells. (C) Reanalysis of cell populations in panel B shows that the C133+ cells (red) are 0.8% of the EpCAM+ population.


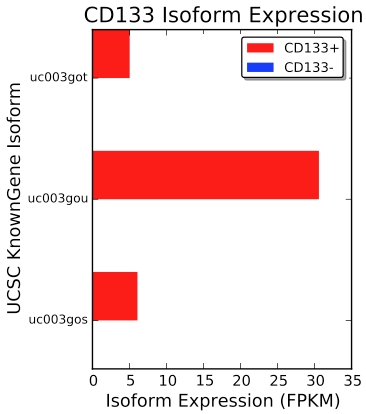


**Figure S2: CD133 (PROM1) isoform expression levels determined from RNA-seq experiments.** Expression of all three CD133 isoforms was observed in the CD133+ subpopulation and undetected in the CD133- subpopulation.


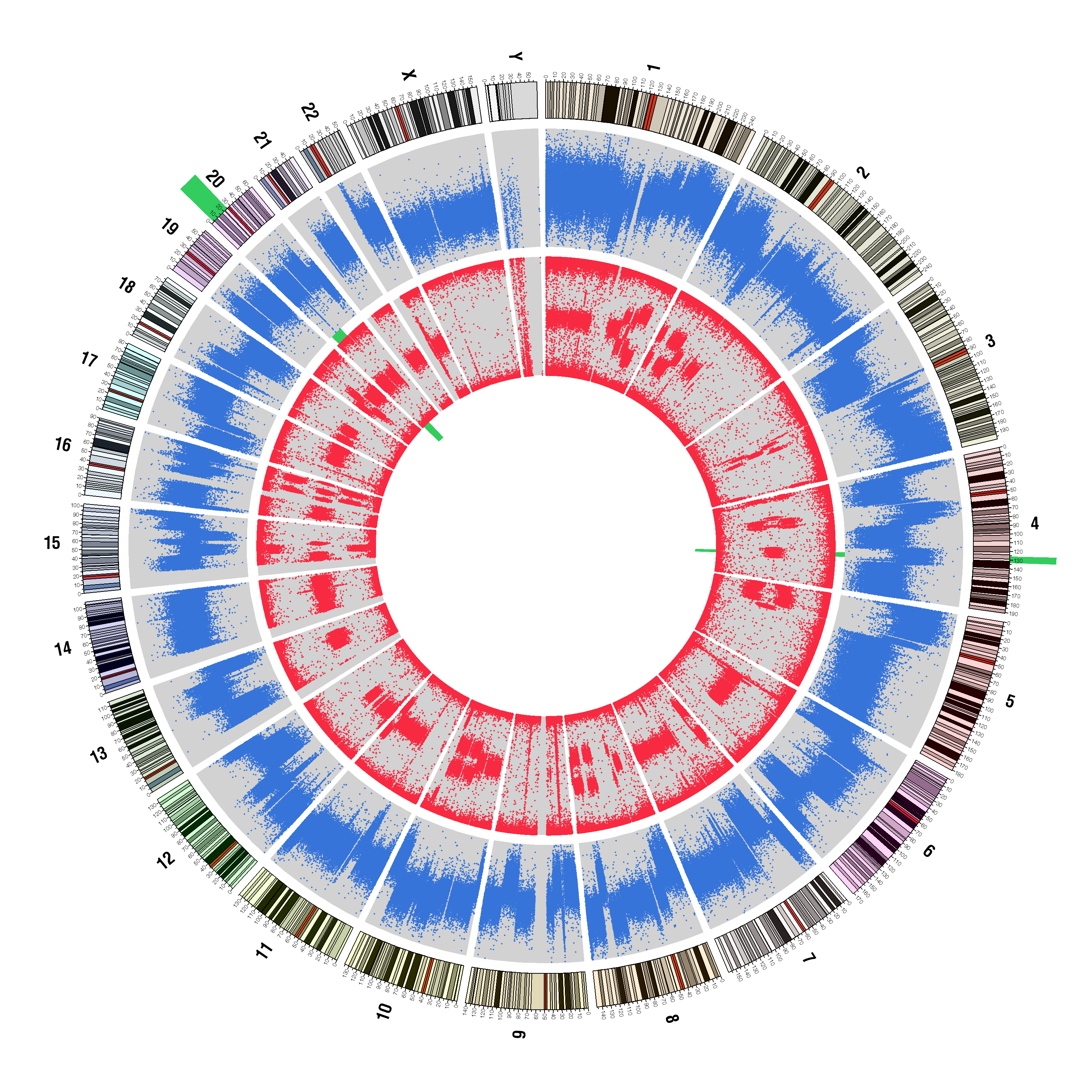


**CD133+**

**
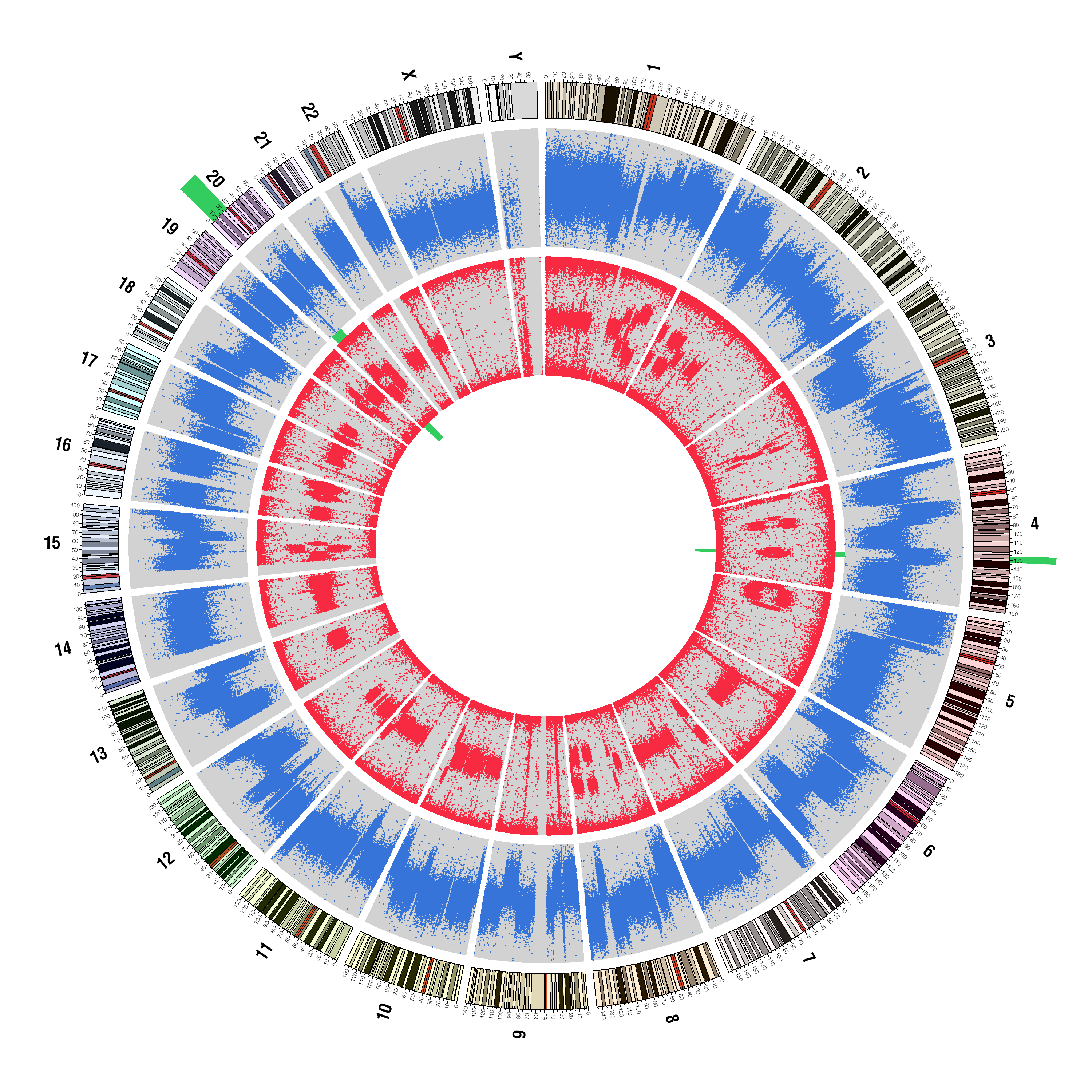
**

**CD133-**

**
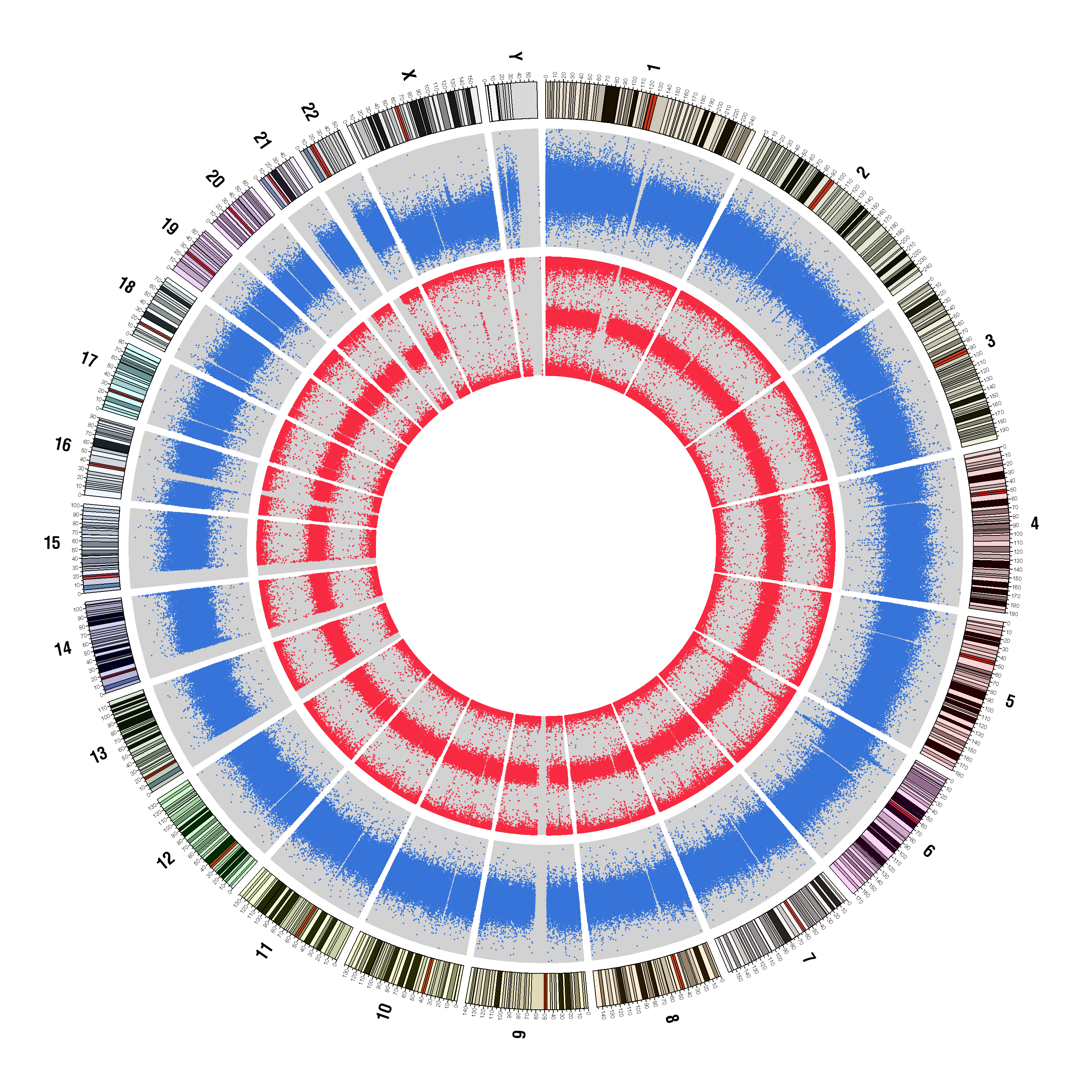
**

**gDNA**

**Figure S4: Chromosome structure analysis by SNP Array.** We hybridized PBMC (gDNA), CD133- and CD133+ genomic DNA to Illumina Human1M-Duo BeadChips. Chromosome ideograms are shown around the outer ring and are oriented pter–qter in a clockwise direction with centromeres indicated in red. Other tracks contain somatic alterations calculated with LogR-ratio (outermost plot, blue) or calculated with B-allele frequency (innermost plot, red). We found that approximately 50% of the tumor cell genomes had undergone copy number changes relative to the germline genome. Within the error range of the array the CD133- and CD133+ genomes are identical (potential regions of difference are indicated in green).


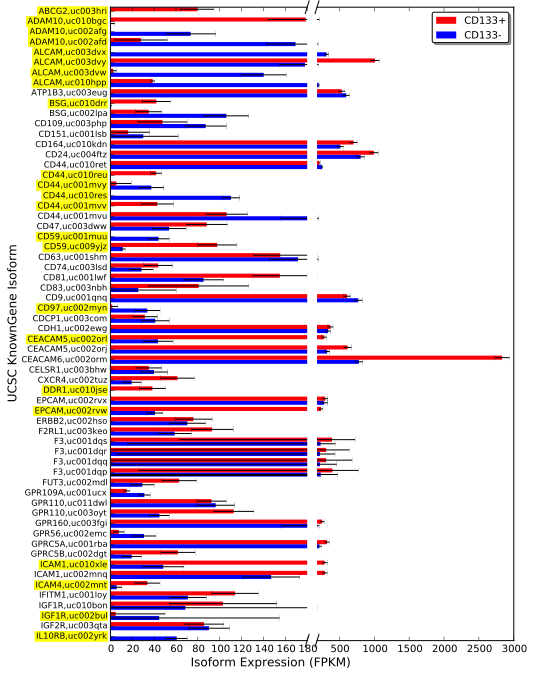


**Figure S5A: Isoform expression levels for CD Molecule, GPCR and ion channel genes** **in CD133+ and CD133- subpopulations – shown alphabetically.** Isoforms significantly differentially expressed greater than four-fold are highlighted in yellow. Error bars are the 95% confidence intervals for the mathematical expression level estimates. Here we use HGNC-approved gene symbols.


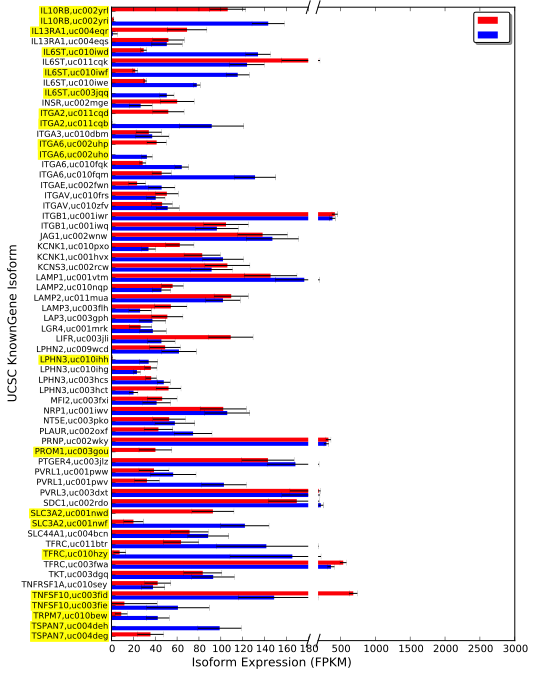


**Figure S5B: Isoform expression levels for CD Molecule, GPCR and ion channel genes** **in CD133+ and CD133- subpopulations – shown alphabetically.** Isoforms significantly differentially expressed greater than four-fold are highlighted in yellow. Error bars are the 95% confidence intervals for the mathematical expression level estimates. Here we use HGNC-approved gene symbols.


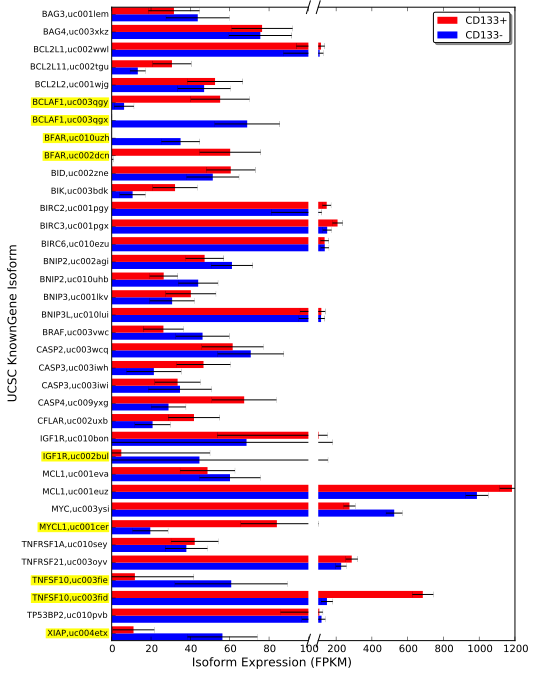


**Figure S6: Isoform expression levels of apoptosis-related genes in CD133+ and CD133- subpopulations.** Isoforms significantly differentially expressed greater than four-fold are highlighted in yellow. Error bars are the 95% confidence intervals for the mathematical expression level estimates. Here we use HGNC-approved gene symbols.


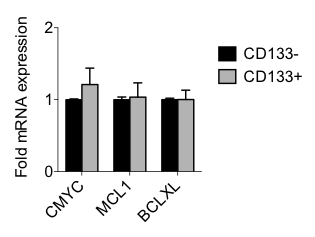


**Figure S7:** RT-qPCR analysis confirms that CMYC, MCL1, and BCLXL are expressed at high levels in both CD133+ and CD133- subpopulations.


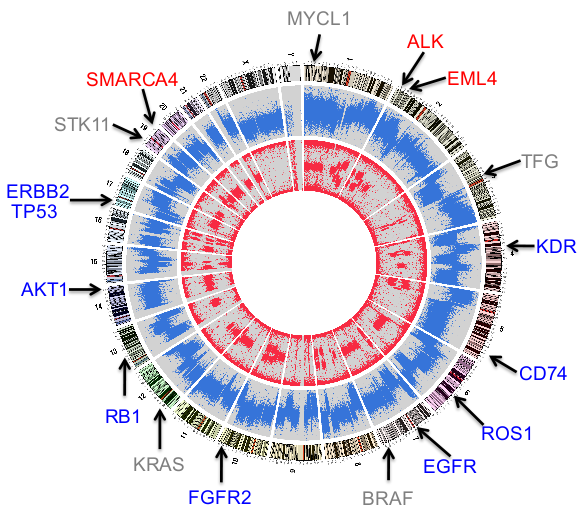


**Figure S8: CNAs in the CD133+ subpopulation.** Chromosome ideograms are shown around the outer ring and are oriented pter–qter in a clockwise direction with centromeres indicated in red. Other tracks contain somatic alterations calculated with LogR-ratio (outermost plot, blue) or calculated with B-allele frequency (innermost plot, red). The genomic locations of 17 genes frequently mutated in lung cancer are shown and indicated if deleted (blue), amplified (red) or copy number neutral (grey) in the studied tumor.

**References**

1. Pinto D, Darvishi K, Shi X, et al. Comprehensive assessment of array-based platforms and calling algorithms for detection of copy number variants. Nat Biotechnol. 2011;29(6):512–20.

2. Weir BA, Woo MS, Getz G, et al. Characterizing the cancer genome in lung adenocarcinoma. Nature. 2007;450(7171):893–8.

3. Bamford S, Dawson E, Forbes S, et al. The COSMIC (Catalogue of Somatic Mutations in Cancer) database and website. Br J Cancer. 2004;91(2):355–8.

4. DePristo MA, Banks E, Poplin R, et al. A framework for variation discovery and genotyping using next-generation DNA sequencing data. Nat Genet. 2011;43(5):491–8.

5. Picard command line tools[Internet]. Available from: http://picard.sourceforge.net

6. Koboldt DC, Zhang Q, Larson DE, et al. VarScan 2: Somatic mutation and copy number alteration discovery in cancer by exome sequencing. Genome Res. 2012;22(3):568–576.

7. Politi K, Lynch TJ. Two Sides of the Same Coin: EGFR Exon 19 Deletions and Insertions in Lung Cancer. Clin Cancer Res. 2012;181490–1492.

8. TCGA[Internet]. Available from: http://cancergenome.nih.gov
